# Aging and cell expansion enhance microRNA diversity in small extracellular vesicles produced from human adipose-derived stem cells

**DOI:** 10.1007/s10616-024-00675-6

**Published:** 2024-12-10

**Authors:** Toshiya Tsubaki, Ryota Chijimatsu, Taiga Takeda, Maki Abe, Takahiro Ochiya, Shinsaku Tsuji, Keita Inoue, Tokio Matsuzaki, Yasuhide Iwanaga, Yasunori Omata, Sakae Tanaka, Taku Saito

**Affiliations:** 1 Orthopaedic Surgery, Sensory and Motor System Medicine, Graduate School of Medicine, The University of Tokyo, 7-3-1 Hongo, Bunkyo-Ku, Tokyo, 113-8655 Japan; 2 Bone and Cartilage Regenerative Medicine, The University of Tokyo Hospital, 7-3-1 Hongo, Bunkyo-Ku, Tokyo, 113-8655 Japan; 3 Department of Chemistry and Biotechnology, Graduate School of Engineering, The University of Tokyo, 7-3-1 Hongo, Bunkyo-Ku, Tokyo, 113-8655 Japan; 4 Center for Comprehensive Genomic Medicine, Okayama University Hospital, 2-5-1, Shikada-Chou, Kita-Ku, Okayama, 700-8558 Japan; 5 Department of Molecular and Cellular Medicine, Tokyo Medical University, 6-7-1 Nishi-Shinjuku, Sinjuku-Ku, Tokyo, 160-0023 Japan; 6 CPC Corporation, 3-4 Kanda Surugadai, Chiyoda-ku, Tokyo, 101-0062 Japan

**Keywords:** Adipose-derived stem cells, Extracellular vesicles, microRNA, Regenerative therapy

## Abstract

Adipose-derived stem cells (ASCs) and their small extracellular vesicles (sEVs) hold significant potential for regenerative medicine due to their tissue repair capabilities. The microRNA (miRNA) content in sEVs varies depending on ASC status; however, the effects of aging and cell passage on miRNA profiles remain unclear. In this study, we examined the effects of donor age and cell expansion on ASC characteristics and transcriptome using ASCs obtained from three young and three old donors. Cell expansion significantly impaired stem cell properties, notably reducing proliferation and differentiation capacities. In contrast, donor age had minimal effects on ASCs. RNA sequencing (RNA-seq) revealed differences in gene expression related to stemness, phagocytosis, and metabolic processes influenced by cell expansion. To investigate miRNA variability, we performed small RNA-seq on sEVs collected from ASCs of all six donors. The miRNA profiles were influenced by donor age and cell passage. Interestingly, functional enrichment analysis indicated that advanced donor age and increased cell passage may enhance the production of miRNAs associated with organ development through various pathways. These findings suggest that donor age and cell expansion differentially influence ASC characteristics and sEV miRNA content, highlighting the need for disease-specific conditioning of ASCs to optimize the therapeutic effects of sEVs in clinical applications.

**Supplementary Information:**

The online version contains supplementary material available at 10.1007/s10616-024-00675-6.

## Introduction

Mesenchymal stem cell (MSC)-based therapies have demonstrated efficacy in treating a wide range of diseases due to their exceptional immunomodulatory capabilities and differentiation potential (Mathiasen et al. 2015; Bloor et al. 2020; Fan et al. 2020; Petrou et al. 2020). Among MSCs, adipose-derived stem cells (ASCs), which can be isolated from abdominal subcutaneous tissue, are less invasive and have superior proliferative potential compared to other MSCs (Dai et al. 2016). ASCs have been used in various clinical trials and applications (Chu et al. 2019). One notable therapeutic effect of ASCs is the secretion of small extracellular vesicles (sEVs), microvesicles, apoptotic bodies, and various cytokines (Fan et al. 2020). sEVs are known to contain nucleic acids such as mRNA and microRNA (miRNA), as well as proteins, which are believed to mediate intercellular interactions and therapeutic effects (Valadi et al. 2007; Kalluri et al. 2020). Their efficacy has been demonstrated in several animal studies (Cho et al. 2018; Bonafede et al. 2020; Takeuchi et al. 2021). Although sEVs are considered relatively safe due to the absence of cells, inconsistencies in their generation and composition present a significant barrier to clinical application.

Several challenges exist in the therapeutic application of sEVs, including difficulties in producing them in large quantities (Han et al. 2023). This issue necessitates the use of cells from diverse donors and their expansion. Regarding the properties of stem cells, there is increasing evidence that age-related changes in tissue stem cells contribute to the breakdown of tissue homeostasis (Micco et al. 2021; López-Otín et al. 2023). Donor age has been shown to affect the proliferation and multipotency of ASCs (Kawagishi-Hotta et al. 2017; Park et al. 2021). However, our understanding of MSC properties and their relationship with the sEVs they secrete remains limited.

In this study, we aimed to elucidate how donor age and cell culture expansion affect the properties of ASCs and the components of sEVs derived from them. We investigated MSC characteristics, including self-renewal ability in monolayer culture, MSC surface marker expression, and differentiation potential into osteogenic, chondrogenic, and adipogenic lineages in vitro (Dominici et al. 2006). We analyzed mRNA expression profiles of ASCs and miRNA components of their sEVs using RNA-seq and small RNA-seq, respectively. We further performed functional enrichment analysis using small RNA-seq data and clarified how donor age and cell proliferation of ASCs affect the properties of miRNA in sEVs.

## Materials and methods

### ASC isolation and cell culture

Human subcutaneous adipose tissues from six donors (three in their 20 s and three in their 70–80 s) were procured through plastic surgery, following a protocol approved by the ethics committee of the University of Tokyo (Institutional Review Board 12008). The donor information is detailed in Table 1. ASCs were isolated as previously described (Chijimatsu et al. 2022). In brief, the harvested adipose tissues were placed on nonwoven fabrics coated with hydroxyapatite and immersed in culture medium. After several days, ASCs that had migrated into the nonwoven fabrics were collected using trypsin. The isolated cells were cultured in MSC NutriStem® XF Basal Medium (NutriStem, Sartorius Stedim Biotech, Lower Saxony, Germany) supplemented with 2% fetal bovine serum (FBS) and 0.1% penicillin–streptomycin at 37 °C in an incubator with humidified 5% CO_2_.

**Table 1:** Donor information

Group	Donor	Sex	Age
Young	1	Male	26
Young	2	Male	25
Young	3	Female	26
Old	4	Male	79
Old	5	Female	80
Old	6	Male	73

### Cell proliferation

ASCs were seeded at a density of 5.0 × 10^5^ cells on 15 cm cell culture dishes. Cell counts were obtained at 80–90% confluency using the LUNA-FL™ (Logos Biosystems, Anyang, Gyeonggi-do, South Korea). The proliferation rate was calculated using the following formula:

Proliferation rate = (cell count at harvest—cell count at seeding) / culture days.

### Differentiation-Induction of adipogenesis

Cells were seeded at a density of 1 × 10^5^ cells/cm^2^ and cultured overnight in 10% FBS-Dulbecco’s Modified Eagle’s Medium (DMEM). Subsequently, the cells were cultured in 10% FBS-DMEM supplemented with 10 µg/mL insulin (Fujifilm Wako Pure Chemical, Osaka, Japan), 200 µM indomethacin (Fujifilm Wako Pure Chemical, Osaka, Japan), 1 µM dexamethasone (Nacalai Tesque, Kyoto, Japan), and 500 µM 3-isobutyl-1-methylxanthine (Fujifilm Wako Pure Chemical, Osaka, Japan) for one week. Cells cultured in 10% FBS-DMEM were used as controls. For lipid droplet detection, cells were fixed with 10% Formalin Neutral Buffer Solution (Fujifilm Wako Pure Chemical, Osaka, Japan), washed with water, and stained with 60% Oil Red O solution (Muto Pure Chemicals, Tokyo, Japan). The stained dye was eluted with 100% isopropanol (Nacalai Tesque, Kyoto, Japan), and absorbance was measured using a microplate reader (Thermo Fisher Scientific, Waltham, Massachusetts, USA) at an excitation wavelength of 515 nm.

### Differentiation-Induction of osteogenesis

Cells were seeded at a density of 5 × 10^4^ cells/cm^2^ and cultured overnight in 10% FBS-DMEM. Subsequently, the cells were cultured in 10% FBS-DMEM supplemented with 10 mM β-glycerophosphate (Sigma–Aldrich, St. Louis, MO, USA), 10 nM dexamethasone (Nacalai Tesque, Kyoto, Japan), and 50 µg/mL ascorbic acid 2-phosphate (Sigma–Aldrich, St. Louis, MO, USA) for three weeks. Cells cultured in 10% FBS-DMEM were used as controls. Differentiation was confirmed by assessing calcium deposition. For analysis, cells were fixed with 10% Formalin Neutral Buffer Solution and washed with water. Calcium deposition was detected following incubation with 1% Alizarin Red S solution (pH 6.4) (Muto Pure Chemicals, Tokyo, Japan). The colored area of the wells was measured using ImageJ software.

### Differentiation-Induction of chondrogenesis

To obtain cell-aggregated pellets, 3 × 10^5^ cells were centrifuged in a 15 ml tube and cultured in 10% FBS-DMEM. The next day, the medium was replaced with a chondrogenic medium comprising high-glucose DMEM containing 110 µg/mL sodium pyruvate (Thermo Fisher Scientific, Waltham, MA, USA) supplemented with 0.2 mM ascorbate-2-phosphate (Sigma–Aldrich, St. Louis, MO, USA), 40 µg/mL L-proline (Fujifilm Wako Pure Chemical), 10 nM dexamethasone (Sigma–Aldrich, St. Louis, MO, USA), 1% ITS + Premix (Corning: 6.25 µg/mL insulin, 6.25 µg/mL transferrin, 6.25 µg/mL selenious acid, 1.25 µg/mL bovine serum albumin, and 5.35 µg/mL linoleic acid), 10 ng/mL transforming growth factor-β3 (TGFβ3) (Peprotech, Rocky Hill, NJ, USA), 20 ng/mL bone morphogenic protein 2 (BMP2) (Medtronic, Dublin, Ireland), and 0.25 nM TD-198946 (Chijimatsu et al. 2019). For assessment, the chondrogenic pellets were fixed with 10% Formalin Neutral Buffer Solution and embedded in paraffin wax. The 4 µm sections were then stained with safranin-O (Waldeck GmbH & Co. KG, Münster, Germany)/fast green (Fujifilm Wako Pure Chemical, Osaka, Japan)/hematoxylin (Muto Pure Chemicals, Tokyo, Japan) staining dye. The positively stained area was measured using QuPath v0.4.3.

### Flow cytometry

Cells were incubated for 30 min at 4 °C with the following fluorescence-conjugated antibodies: PE-conjugated anti-hCD73 (BioLegend, San Diego, CA, USA, 344003), FITC-conjugated anti-hCD90 (BioLegend, San Diego, CA, USA, 328107), APC-conjugated anti-hCD105 (BioLegend, San Diego, CA, USA, 323207), PE-conjugated anti-hCD34 (BioLegend, San Diego, CA, USA, 343505), and APC-conjugated anti-hCD45 (BioLegend, San Diego, CA, USA, 368511). Isotype control antibodies were used as negative controls (BioLegend, San Diego, CA, USA, 400132, 400121, and 400111). The cells were washed with 0.1% BSA-PBS and resuspended for analysis with CytoFLEX S (Beckman Coulter, Brea, CA, USA) according to the manufacturer’s protocol. Data from sorting were analyzed using CytoExpert software (Beckman Coulter, Brea, CA, USA).

### Cellular senescence detection

Senescent-associated β-galactosidase (SA β-gal) staining was conducted according to the manufacturer’s instructions (Dojindo Laboratories, Kumamoto, Japan). Staining intensity was measured using a microplate reader at an excitation wavelength of 520 nm and an emission wavelength of 565 nm for SA β-gal staining, and at an excitation wavelength of 350 nm and an emission wavelength of 461 nm for cell count staining. SA β-gal activity was calculated using the following formula: SA β-gal activity = SPiDER-βGal fluorescence intensity / (cell count fluorescence intensity—cell count blank fluorescence intensity).

### RNA-seq

Total RNA was extracted using the Direct-zol RNA kit (Zymo Research, Irvine, CA, USA) according to the manufacturer’s procedure. All samples were confirmed to have an A260/A280 ratio of 1.8–2.0. Library preparation was performed using the Strand-specific library preparation method (dUTP method). Sequencing was performed using the Illumina NovaSeq 6000 (Illumina, San Diego, CA, USA) with 150-bp paired-end reads.

### sEV extraction

ASCs were seeded into 15-cm dishes and cultured to 80% confluence. The medium was then replaced with sEV collection medium (NutriStem:DMEM = 1:1). After 48 h, the culture supernatant was collected. The supernatant was centrifuged at 2000 g for 10 min to remove cell debris and filtered using a 0.22 µm filter (Thermo Fisher Scientific, Massachusetts, USA). The filtered supernatant was ultrafiltered using Vivaspin®20 Membrane: 100,000 MWCO PES (Sartorius Stedim Biotech, Lower Saxony, Germany). sEVs were purified from the ultrafiltered supernatant using Exoquick TC (System Biosciences, Palo Alto, CA, USA).

### sEV validation-Enzyme-linked immunosorbent assay (ELISA)

CD9 and CD63, surface markers of sEVs, were analyzed using ELISA according to the manufacturer’s instructions (Cosmo Bio Co., Ltd., Tokyo, Japan). The plate was coated with anti-human CD9 antibody. Upon adding the sample, sEVs bearing CD63 on their surface were trapped. HRP-conjugated anti-human CD63 antibody was then reacted with CD63 on the trapped sEVs, and after substrate addition, colorimetric quantification was performed using a microplate reader at a measurement wavelength of 450 nm.

### sEV validation-Nanoparticle tracking analysis

The particle size distribution was measured using the NanoSight LM10 (Quantum Design Japan, Inc., Tokyo, Japan). In brief, after removing debris through centrifugation and filtration, the culture medium was diluted six times for analysis. The following parameters were used for imaging: Camera level 13, detection threshold 7, and screen gain 4.

### Small RNA-seq

The precipitated sEVs (sEV extraction) were lysed with TRI reagent (Molecular Research Center, Inc., Cincinnati, OH, USA), and RNA was extracted using the Direct-zol RNA kit. Small RNA-seq was performed using DNBSEQ (Mgi Tech Co., Ltd., Shenzhen, GD, China) with 50-bp single-end reads.

### Bioinformatics analysis-ASC mRNA

Raw data were mapped to a reference genome from GENCODE (GRCh38.primary_assembly.genome.fa and gencode.v41.annotation.gtf) using STAR v2.7.10a (Dobin et al. 2013) after removing adapter sequences with fastp v0.20.1 (Chen et al. 2018a, b). Expression levels were calculated using RSEM v1.3.3 (Li et al. 2011). Low-expression genes were filtered out, and the remaining counts were normalized using DESeq2 (Love et al. 2014). Heatmaps were visualized with pheatmap v1.0.12. In the five-group analysis, differentially expressed genes (DEGs) and their rankings were determined using TCC v1.42.0 (Sun et al. 2013) and EBSeq v2.0.0 (Leng et al. 2022). Gene Ontology (GO) analyses were performed using clusterProfiler v4.10.1, with a p-value cutoff of < 0.005 for enrichment terms. Bar plots of log10(p-value) values were marked as positive or negative according to the sample and illustrated using ggplot2 (Wickham 2009).

### Bioinformatics analysis-sEV miRNA

The raw data were mapped to a reference genome (GCF_000001405.38_GRCh38.p12) using Bowtie2 v2.2.9 (Langmead et al. 2012) after adapter sequences were removed using SOAPnuke v1.5.6 (Chen et al. 2018a, b). Expression values were calculated using the following formula: Expression = (the number of UMI reads that are mapped to the small RNA * 1,000,000) / the UMI number of total clean reads in the sample. Heatmaps were visualized using pheatmap v1.0.12. For miRNA target prediction, multiMiR v1.24.0 (Ru et al. 2014) was utilized, with target genes identified as those predicted by all three tools: miRanda, miRDB, and TargetScan. Functional prediction of target genes was performed using clusterProfiler v4.10.1 for enrichment analysis. For disease-related genes, the dataset from Enrichr (Kuleshov et al. 2016) was used. The Disease_Signatures_from_GEO_up_2014 dataset lists genes that are upregulated in each disease based on the data submitted to the Gene Expression Omnibus (GEO).

### Statistics

R 4.3.3 (R Core Team. 2024) was used for statistical analysis. An unpaired t-test or Wilcoxon rank-sum test was applied to compare two groups. For multiple comparisons, statistical analysis was performed using one-way analysis of variance (ANOVA) and Tukey’s honestly significant difference test. Pearson correlation tests were performed to calculate correlations. A p-value of < 0.05 was considered significant.

## Results

### Stem cell properties of young and old donor-derived ASCs in the early stage of in vitro cell expansion

**Fig. 1:**
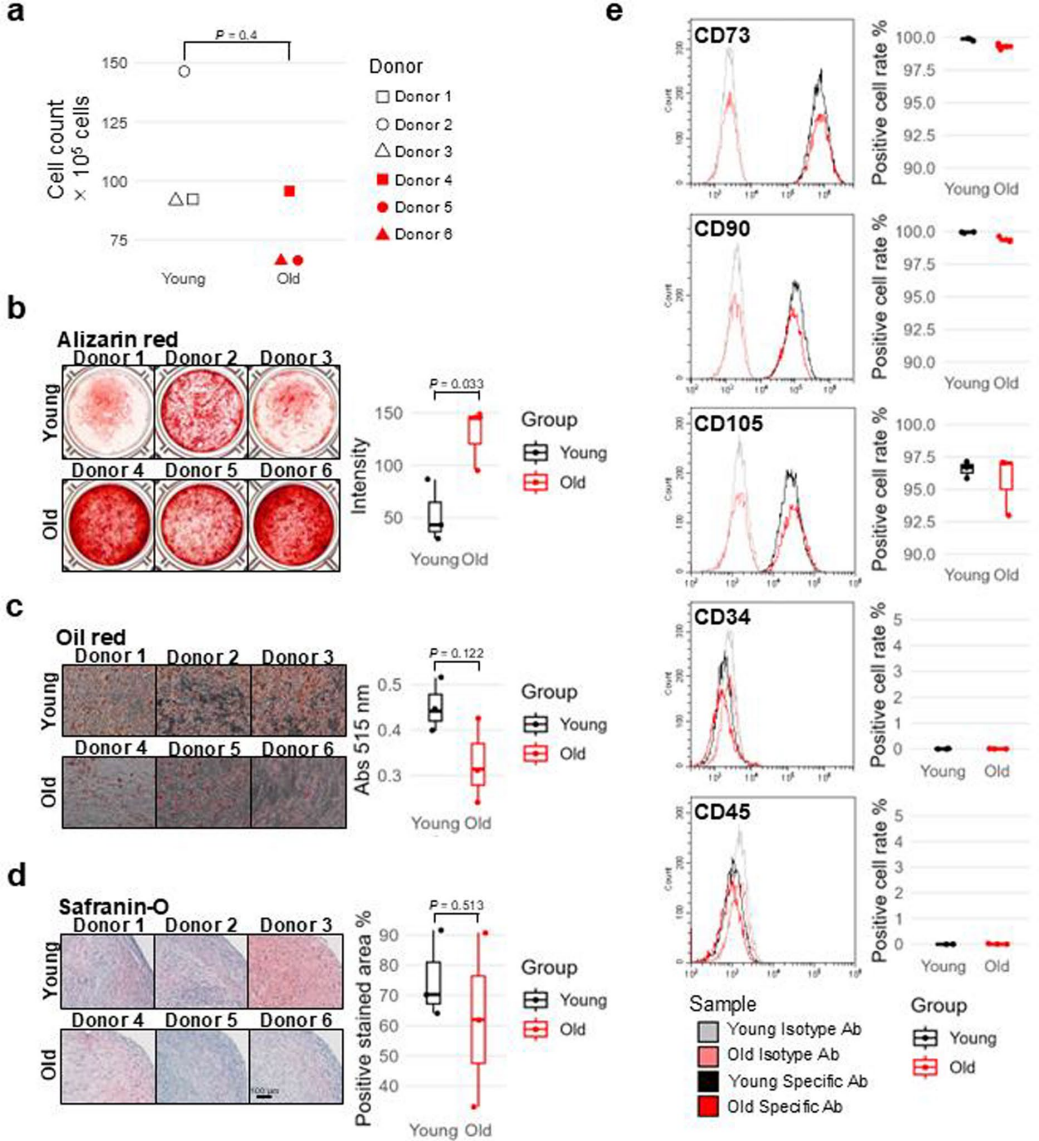
Stem cell properties of young and old donor-derived ASCs in the early stage of in vitro cell expansion. **a** Cell counts of ASCs derived from three young and three old donors four days after seeding. Statistical significance was determined using the Wilcoxon rank**-**sum test. **b** Alizarin red staining of ASCs after osteogenic differentiation induction. The right box plot shows stain intensity. **c** Oil red staining of ASCs after adipogenic differentiation induction. The right box plot shows the absorbance of the eluted dye. **d** Safranin-O staining of ASCs after chondrogenic differentiation induction. The right box plot shows the percentage of the positive area. **b–d** ASCs from three young and three old donors were used. Statistical significance was determined using an unpaired t-test. **e** Flow cytometry of ASCs from three young and three old donors using antibodies for MSC positive and negative markers. The left panels show representative data from Donor 1 (young) and Donor 4 (old), and the right box plots show the percentage of cells positive for each surface antigen marker

We first analyzed the MSC-defining characteristics of ASCs derived from three young and three elderly donors. ASCs from both young and old donors showed similar proliferative capacities, showing no difference in cell count on the fourth day after seeding (Fig. 1a). In terms of differentiation capacity, osteogenic differentiation, as determined by alizarin red staining, was significantly increased in aged MSCs (Fig. 1b). Although there was a trend toward reduced lipid droplet formation with aging, oil red staining did not show significant differences in adipogenic differentiation (Fig. 1c). Chondrogenic differentiation did not differ significantly between young and old donors (Fig. 1d). Similarly, both young and old donor groups exhibited similar expression levels of stem cell markers and negative markers (Fig. 1e). These data suggest that there were no obvious differences in proliferative capacity and MSC marker expression among young and old donor-derived ASCs in the early stage of in vitro expansion, although differentiation capacities varied.

### Stem cell properties and senescence of young and old donor-derived ASCs in the late stage of in vitro cell expansion

**Fig. 2:**
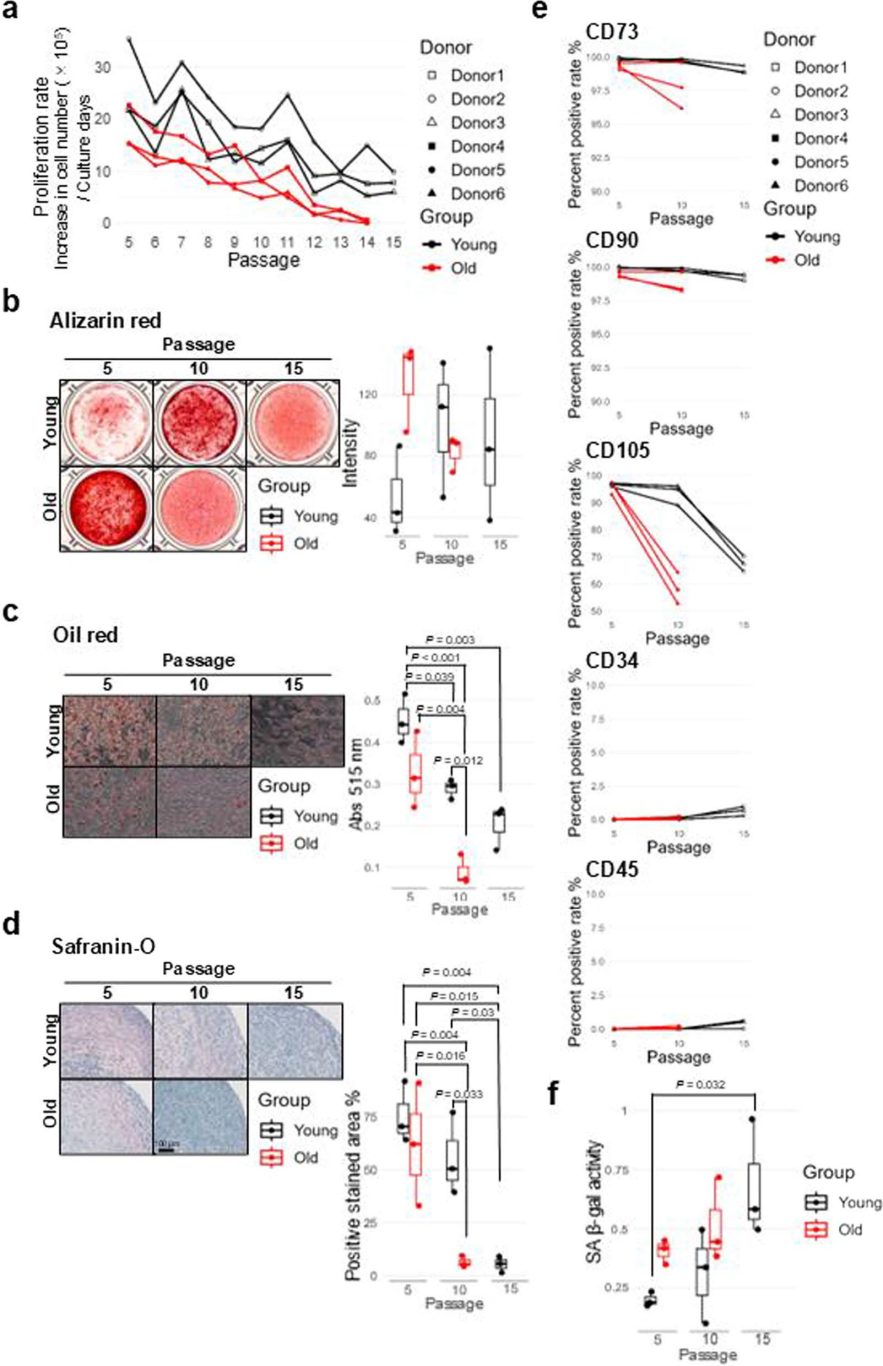
Stem cell properties and senescence of young and old donor-derived ASCs in the late stage of in vitro cell expansion. **a** Cell proliferation rates of ASCs derived from three young and three old donors at each passage. **b** Alizarin red staining of ASCs after osteogenic differentiation induction. The right box plot shows stain intensity. **c** Oil red staining of ASCs after adipogenic differentiation induction. The right box plot shows the absorbance of the eluted dye. **d** Safranin-O staining of ASCs after chondrogenic differentiation induction. The right box plot shows the percentage of the positive area. **b–d** ASCs from three young and three old donors at each passage were used. Representative images from the three replicates are shown. Statistical significance was determined using one-way ANOVA with post-hoc Tukey’s honestly significant difference test. **e** Time course of positive rates for MSC positive and negative markers in ASCs from six donors, determined by flow cytometry. **f** SA β-Gal activity of ASCs. ASCs from three young and three old donors at each passage were used. Statistical significance was determined using one-way ANOVA with post-hoc Tukey’s honestly significant difference test

Next, we investigated the effects of cell expansion on the stem cell properties of ASCs. Generally, long-term primary cultured cells exhibit cellular senescence. At passage 5, both young and old donor-derived ASCs showed similar proliferation rates (Fig. 1a). However, old donor-ASCs exhibited a rapid decline in proliferation rates thereafter and nearly stopped proliferating around passages 13–14 (Fig. 2a). In contrast, young donor-ASCs continued to proliferate until passage 15 (Fig. 2a). Osteogenic differentiation capacities of both groups did not change significantly with cell expansion (Fig. 2b). Meanwhile, adipogenic and chondrogenic differentiation capacities decreased with passages (Fig. 2c,d). At passage 5, there were no significant differences in these capacities between young and old donor-ASCs (Fig. 2c,d). However, at passage 10, these capacities were significantly reduced in old donor-ASCs (Fig. 2c,d). Interestingly, the differentiation capabilities of Young-Passage-10 samples were similar to those of Old-Passage-5 and Old-Passage-10 samples, and the differentiation capabilities of Young-Passage-15 samples were more similar to those of Old-Passage-10 samples (Fig. 2b–d). Consistent with these findings, stem cell marker analysis showed an increased population of CD105-negative cells in Young-Passage-15 (10.96–12.53%) and Old-Passage-10 samples (11.33–20.58%) (Fig. 2e). We then assessed cellular senescence under each condition. SA β-gal staining revealed that activity was lowest in Young-Passage-5 samples, followed by Young-Passage-10, Old-Passage-5, Old-Passage-10, and Young-Passage-15 (Fig. 2f). However, there were no significant differences except between Young-Passage-5 and Young-Passage-15 (Fig. 2f). These results imply that cellular senescence is influenced by cell expansion, rather than donor age.

### Effects of donor age and cell expansion on the transcriptome of ASCs

**Fig. 3:**
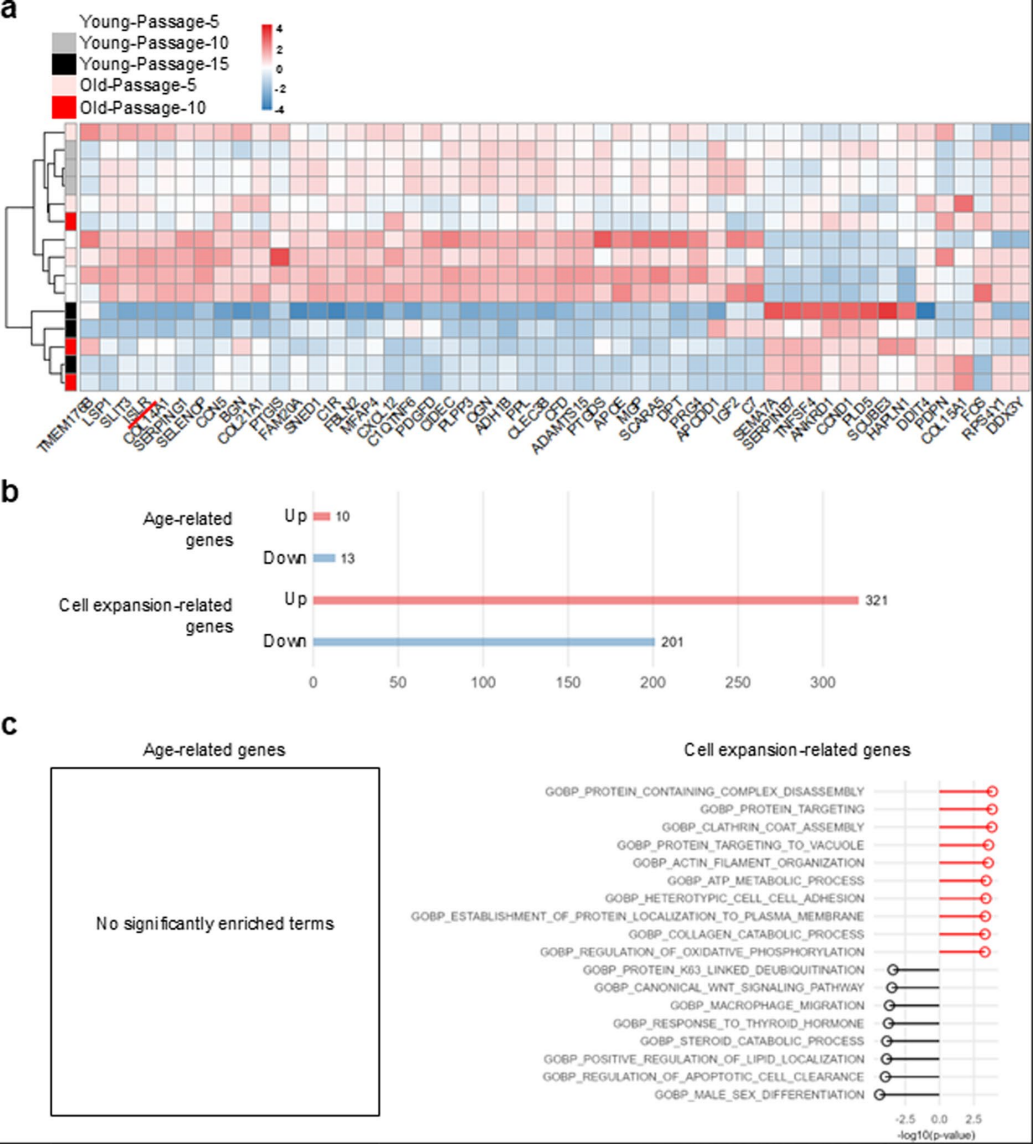
Gene expression changes in ASCs related to aging and cell expansion. **a** Heatmap showing the top 50 differentially expressed genes (DEGs) across five groups of ASCs, each consisting of three biological replicates. Hierarchical clustering was performed using the Ward.D2 method. Columns represent genes, and rows represent samples, with the red underline highlighting *ISLR*. **b** Bar plot showing the number of DEGs identified by EBSeq. The upper portion shows age-related DEGs (23 genes), and the lower portion presents passage-related DEGs (513 genes). The numbers above the bars indicate the DEG count. **c** Bar plot representing the enriched Gene Ontology (GO) terms related to cell expansion-associated DEGs within the “Biological Process” category. The y-axis lists significant GO terms, while the x-axis represents the -log10(p-value). No significant GO terms were identified for age-related DEGs (left box)

Next, we performed RNA-seq on each ASC sample (Young-Passage-5, Young-Passage-10, Young-Passage-15, Old-Passage-5, and Old-Passage-10) to capture molecular changes in ASCs. Figure 3A shows a heatmap of the top 50 DEGs, highlighting the similar expression patterns in Young-Passage-15 and Old-Passage-10 compared to the other three samples. Notably, *ISLR*, a marker of stem cell stemness (Hara et al. 2021), showed decreased expression with both aging and cell expansion (Fig. 3a). Analysis of gene expression patterns revealed a limited number of age-related DEGs (10 upregulated genes and 13 downregulated genes), whereas a large number of DEGs associated with cell expansion were identified (321 upregulated genes and 201 downregulated genes) (Fig. 3b). Further enrichment analysis was conducted on these gene sets. For the aging-related pattern, no GO term was enriched (Fig. 3c). In contrast, for the cell expansion-specific pattern, terms related to metabolic processes, including ATP metabolism and oxidative phosphorylation, were enriched. Conversely, terms associated with stemness, such as Wnt signaling, and those related to phagocytosis, were downregulated by cell expansion (Fig. 3c). Overall, the transcriptome analysis suggested that cell expansion, rather than donor age, negatively affects stem cell properties, consistent with the results of conventional evaluations (Figs. 1, 2).

### Aging and cell expansion enhance diversity of predicted functional roles of miRNA

**Fig. 4:**
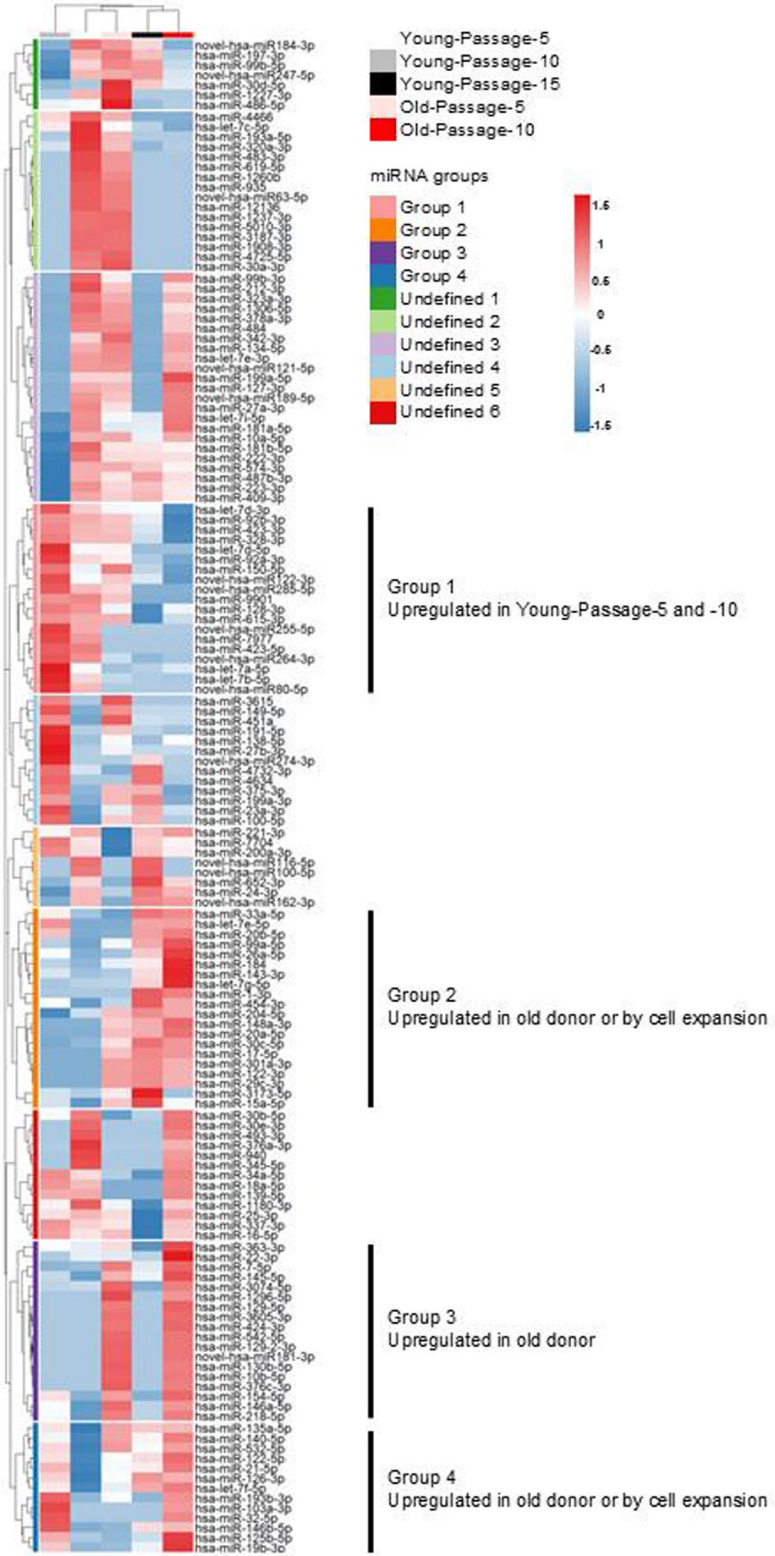
Hierarchical clustering of miRNAs based on small RNA-seq analysis. Heatmap of 150 miRNAs with read counts > 0 in at least two samples across five groups. Hierarchical clustering was performed by the Ward.D2 method. Rows represent individual miRNAs, while columns denote samples. Based on hierarchical clustering, the miRNAs are categorized into ten distinct groups

We then investigated the miRNA signature in sEVs. First, we confirmed the quality of the sEVs collected from the five ASC groups. In all groups, the sizes of the sEVs were within the typical range (40–160 nm) (Supplementary Fig. 1a), and representative sEV markers (Kalluri et al. 2020) were highly expressed (Supplementary Fig. 1b). To examine the miRNA profiles in sEVs across different samples, small RNA-seq was conducted. The small RNA-seq analysis identified 150 miRNAs with read counts > 0 in at least two samples, and hierarchical clustering categorized these miRNAs into ten distinct groups (Fig. 4). Group 1 was enriched in Young-Passage-5 and -10 but significantly decreased with further cell expansion (Fig. 4). Group 3 was uniquely expressed in old donor ASCs (Fig. 4). Groups 2 and 4 exhibited increased expression with aging and cell expansion. However, Group 2 showed increased expression later in the cell expansion process, specifically at Passage 15 in young ASCs, while Group 4 demonstrated earlier upregulation, starting at Passage 10 in young ASCs (Fig. 4).

**Fig. 5:**
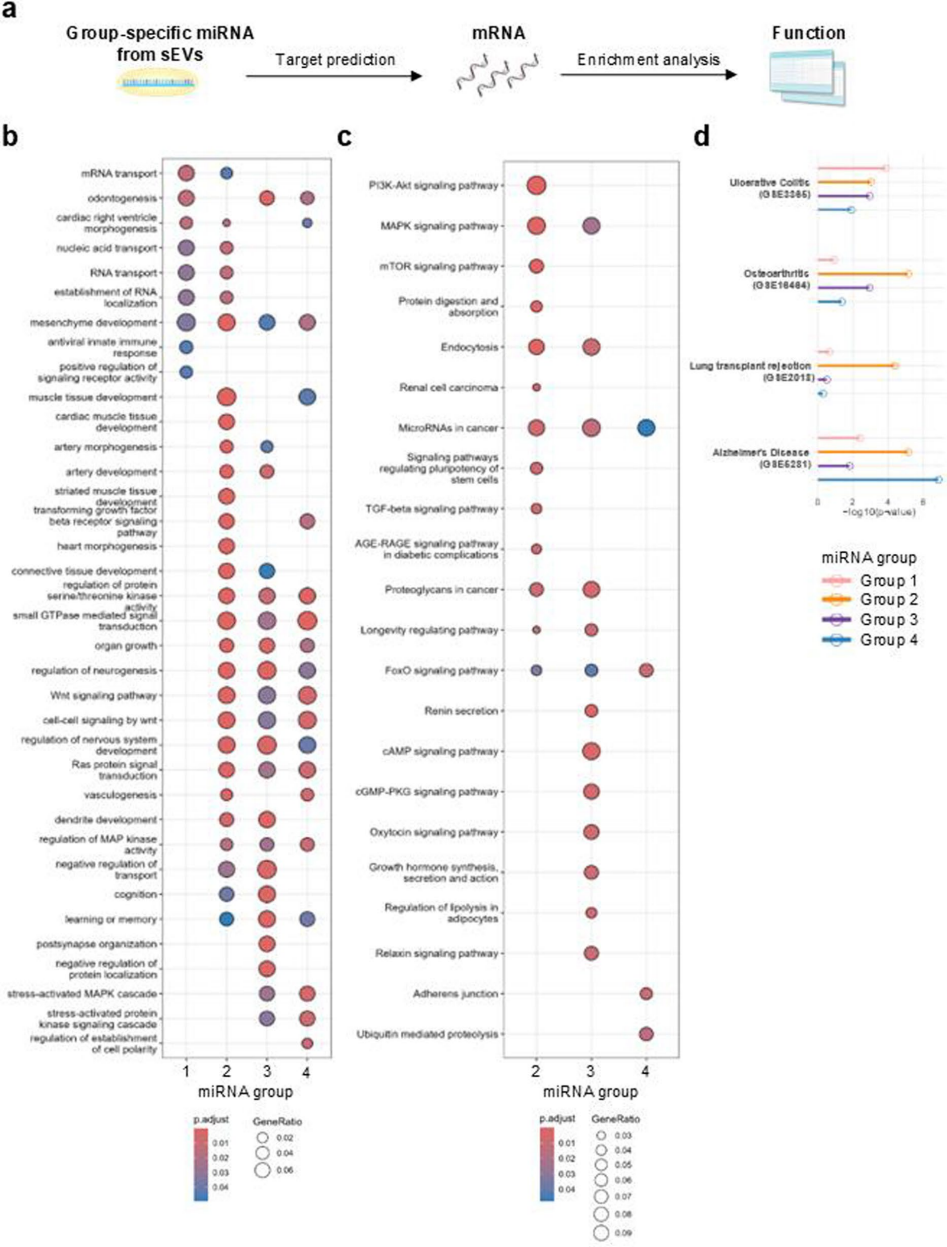
Function prediction of group-specific miRNAs derived from sEVs. **a** Schematic workflow illustrating the analysis of group-specific miRNAs from sEVs, targeting mRNA prediction, and functional analysis. **b–c** Dot plot showing GO **(b)** and KEGG pathway **(c)** enrichment analysis for target mRNAs. The y-axis lists each GO term, while dot size represents the gene ratio, and color indicates the p-value, transitioning from blue (higher p-value) to red (lower p-value). **d** Bar plot of enrichment analysis based on disease-related gene lists. The y-axis represents each disease, and the x-axis is the -log10(p-value). Bar colors correspond to miRNA Groups 1–4

Finally, to infer the functions of the extracted miRNA groups, we predicted their target genes and performed enrichment analysis (Fig. 5a). The top 20 predicted target genes in Groups 1–4 were listed in Table 2. When examining the Biological Process GO terms, Group 1, distinct in Young-Passage-5 and -10, was not characterized by specific terms, except for those related to the immune system, such as the “antiviral innate immune response” (Fig. 5b). On the other hand, Groups 2, 3, and 4, related to old donor or cell expansion, were characterized by diverse terms related to the development of various kinds of organs (Fig. 5b). Notably, terms related to the Wnt signaling pathway were enhanced in these three groups (Fig. 5b). Additionally, enrichment analysis at the pathway level was conducted using KEGG pathways. Signaling pathways related to stem cell properties, homeostasis, and longevity, such as the “signaling pathways regulating pluripotency of stem cells,” “TGF-beta signaling pathway,” “longevity regulating pathway,” and “FoxO signaling pathway” were enriched in Groups 2, 3, and 4 (Fig. 5c). In contrast, no significant pathway was enriched in Group 1. These results suggest that donor age and cell expansion enhance the production of sEVs, which contain a more diverse range of miRNAs, each with distinct functions. Furthermore, considering the clinical applications of sEVs, we conducted an enrichment analysis using the Disease_Signatures_from_GEO_up_2014 database. We filtered the results based on an adjusted p-value of less than 0.05 for at least one miRNA group. Next, we focused on diseases registered in clinical trials as therapeutic targets for MSC-derived extracellular vesicles, including ulcerative colitis, transplant rejection, osteoarthritis, and Alzheimer’s disease (Gimino et al. 2003; Burczynski et al. 2006; Liang et al. 2008; He et al. 2018; Kou et al. 2022; Lotfy et al. 2023; Tan et al. 2024). Interestingly, the number of predicted target genes in each miRNA group varied by disease (Fig. 5d), suggesting that disease-specific conditioning of ASCs may be necessary to optimize the therapeutic effects of sEVs.

**Table 2:** Top 20 predicted mRNAs in each miRNA group

Group 1	Group 2	Group 3	Group 4
BCL2L11	CAPRIN2	BCL2L11	BCL2L11
SLC10A7	RAP2C	NR4A3	CPEB3
NME6	FRS2	EBF1	PLEKHA1
FN1P1	FZD3	RBMS3	BACH1
AHCTF1	RPS6KA5	RPS6KA5	RGL1
ARID3B	UEVLD	SEMA6A	DICER1
BACH1	E2F5	GATA6	PTPRD
BSDC1	ACSL4	KLF4	HIPK3
C8orf58	DYNC1LI2	LRCH1	KRAS
CCNJ	JARID2	RSBN1	YOD1
E2F5	ZBTB18	SHANK2	ARID3B
FIGN	ENPP5	ARID4A	ARMC1
GATM	NABP1	NDFIP2	CCNJ
LRIG3	XRN1	RABEP1	CPEB1
MAP4K3	ZFYVE9	SLC17A6	FBXW7
MDM4	BCL2L11	ZEB2	KLF4
PGRMC1	CPEB1	BZW1	LRIG3
PLEKHB2	PPP1R15B	DCAF6	MAP4K3
PNISR	TBL1XR1	DOCK9	PHTF2

## Discussion

In the present study, we demonstrated that the properties of ASCs change with donor age and cell expansion. Although the stem cell properties of ASCs derived from young and elderly donors were similar in the early stages of cell expansion, many were significantly impaired during cell expansion, accompanied by cellular senescence. The RNA-seq displayed the according transcriptomic changes of ASCs especially by cell expansion. In expanded ASCs, the terms related to stem cell property decreased, while those associated with metabolic processes increased. On the other hand, the expression profiles of miRNA in the sEVs changed by both factors. Notably, miRNA groups enhanced in old and expanded ASCs included a broader range of miRNAs, associated with development of various tissues or organs. Furthermore, these miRNA groups were also predicted to have distinct effects on different diseases. These findings suggest that sEV composition and their potential roles may be influenced by donor age and ASC passage, producing distinct miRNA signatures depending on cellular conditions.

Regarding the functions of miRNAs in MSC-derived sEVs, previous studies have reported the therapeutic potential of several miRNAs, such as miR-125a-3p, miR-494, and let-7b (Nakamura et al. 2015; Ti et al. 2015; Fujii et al. 2018; Pi et al. 2022). When using sEVs for clinical applications, it is essential to comprehensively consider their contents. The current findings from small RNA-seq indicate that the diversity of miRNAs is significantly enhanced by older donor age and increased cell passage. These results suggest that the conditions of ASC culture should be modified depending on the intended purpose. Previous reports have documented the therapeutic effects of MSC-derived sEVs on angiogenesis and neurogenesis (Shabbir et al. 2015; Gong et al. 2017; Yang et al. 2017; Pi et al. 2022). However, in certain pathological conditions, such as cancer and neuropathic pain, angiogenesis and miswiring of neurogenesis may contribute to disease progression (Lugano et al. 2023; Gangadharan et al. 2022). Given these findings, both donor age and ASC expansion should be considered to prepare more effective sEVs for each target disease. We observed large differences in the extent to which miRNA groups from sEVs target genes that are upregulated in diseases such as ulcerative colitis (Burczynski et al. 2006), transplant rejection (Gimino et al. 2003), osteoarthritis (He et al. 2018), and Alzheimer’s disease (Liang et al. 2008). This highlights the importance of selecting effective sEVs tailored for each disease and optimizing ASC conditions. Nonetheless, this study had several limitations, including a lack of replicates in the small RNA-seq analysis, which may limit the assessment of biological variability. Although mRNAs and secreted proteins were also present in sEVs, they were not investigated in this study. Additionally, experimental validation is needed to determine the biological significance of changes in miRNA profiles within sEVs. As a future direction, optimizing the use of sEVs for specific applications will require the development of suitable in vitro evaluation systems and validation of their functionality using in vivo animal models.

The impact of donor age and cell expansion on MSC proliferative and differentiation capacities has been debated. Regarding donor age, some reports suggest no significant changes in proliferation and differentiation capacities, while others indicate a decrease (Wu et al. 2013; Marędziak et al 2016; Kawagishi-Hotta et al. 2017; Liu et al. 2017; Park et al. 2021). In contrast, it is well known that in vitro cell expansion leads to a decline in proliferative capacity, attributed to cellular senescence (Shay and Wright 2000). In old donors, this reduction in proliferation tends to occur at an earlier passage (Stenderup et al. 2003). Concerning differentiation, adipogenic differentiation capacity declines with cell expansion, while the effects on osteogenic differentiation capacity are conflicting (Digirolamo et al. 1999; Stenderup et al. 2003). Our data are consistent with these findings, showing that ASCs from old donors exhibit a more pronounced decline in proliferative capacity with cell expansion compared to those from young donors (Stenderup et al. 2003). Regarding differentiation capacities, ASCs from both young and old donors exhibited no significant changes at the early stage of in vitro cell expansion; however, significant differences emerged as cell passages increased, particularly in adipogenic and chondrogenic differentiation capacities. This trend, where differences become apparent only at the later stages of in vitro cell expansion, represents a novel observation in our study.

Our RNA-seq analysis of ASCs revealed that mRNA expression profiles were influenced by cell expansion rather than donor age. Terms related to oxidative phosphorylation were enriched due to cell expansion, while terms related to phagocytosis and stemness, such as Wnt signaling, were diminished. The metabolic shift toward oxidative phosphorylation in MSCs is linked to in vitro cell proliferation and is associated with the accumulation of reactive oxygen species and cellular senescence (Yan et al. 2021). Furthermore, tissue stem cells have been reported to possess phagocytic activity and play a role in tissue homeostasis (Stewart et al. 2024). The current RNA-seq data are consistent with these previous studies.

In conclusion, our study reveals that donor age and cell passage increase the diversity of miRNAs in sEVs while attenuating the MSC characteristics of the source ASCs. These findings highlight the importance of optimizing ASC conditions to harvest effective sEVs. Further research with larger sample sizes and functional validation is necessary to enhance the clinical application of sEVs derived from ASCs.

## Supplementary Information

Below is the link to the electronic supplementary material.

Supplementary file1 (PDF 179 KB)

## Data Availability

The RNA-seq data of ASCs from young and old donors, small RNA-seq data of sEVs collected from ASC culture supernatants were deposited in the GEO under accession numbers GSE276163, GSE276164, respectively.
