# Supplementary material for: Aging and cell expansion enhance microRNA diversity in small extracellular vesicles produced from human adipose-derived stem cells
Source: Cytotechnology. 2024 Dec 10;77(1):15. doi: 10.1007/s10616-024-00675-6 (PMC11631832; doi:10.1007/s10616-024-00675-6)
Supplement: Supplementary file 1 — Supplementary file1 (PDF 179 KB) [file 10616_2024_675_MOESM1_ESM.pdf]

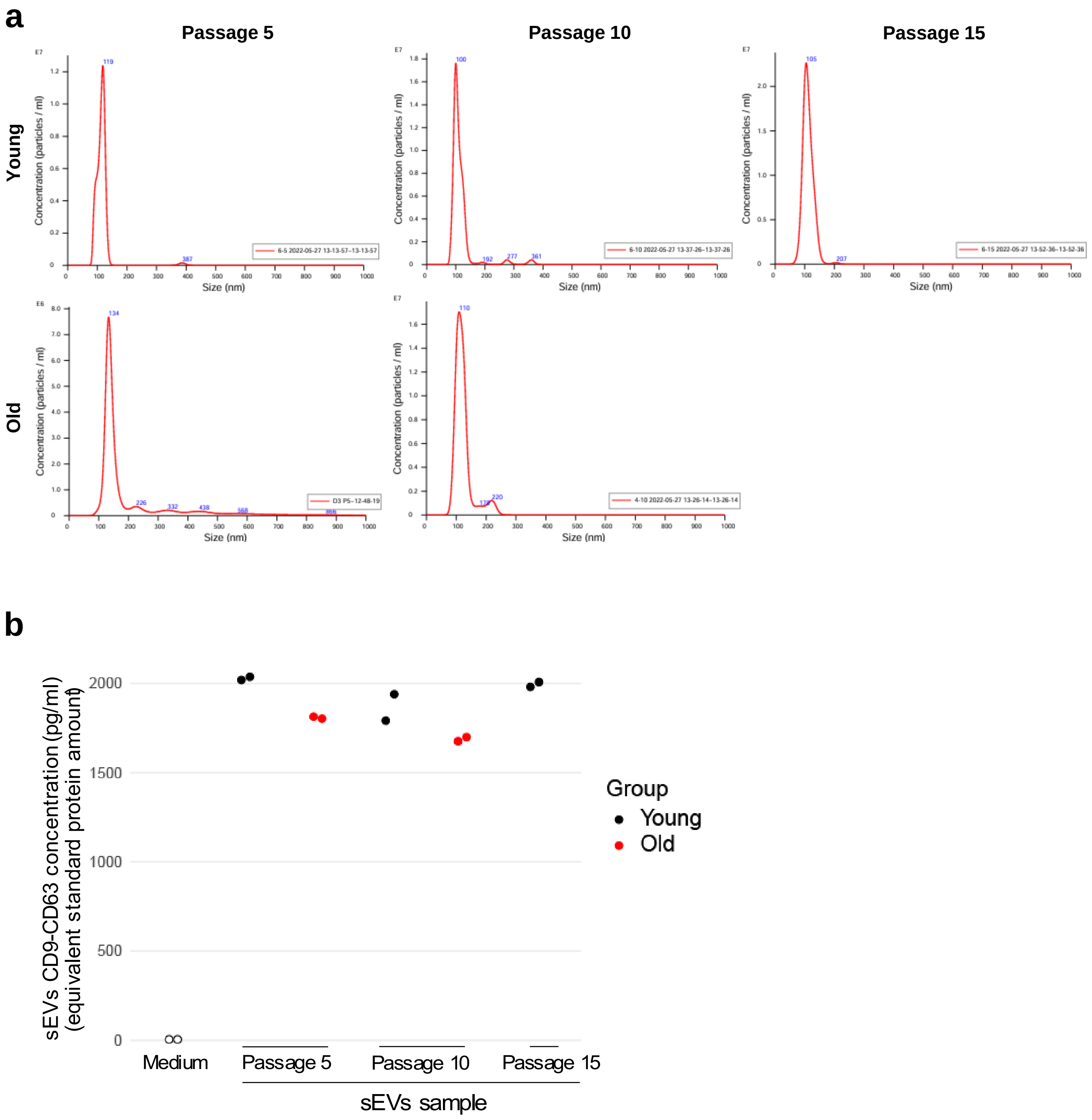

Supplementary Fig. 1 Validation of sEVs. (a) Nanoparticle tracking analysis showing the size distribution of nanoparticles from each sample. The graph displays the size distribution of nanoparticles. Representative graphs from the three replicates are shown. **(b)** ELISA for sEV markers derived from ASCs. The dot plot shows sEVs CD9–CD63 concentration normalized to the standard protein amount from two experiments.
